# Uncovering the Exosomes Diversity: A Window of Opportunity for Tumor Progression Monitoring

**DOI:** 10.3390/ph13080180

**Published:** 2020-08-04

**Authors:** Domenico Maisano, Selena Mimmi, Rossella Russo, Antonella Fioravanti, Giuseppe Fiume, Eleonora Vecchio, Nancy Nisticò, Ileana Quinto, Enrico Iaccino

**Affiliations:** 1 Department of Experimental and Clinical Medicine, University “Magna Graecia” of Catanzaro, 88100 Catanzaro, Italy; mimmi@unicz.it (S.M.); fiume@unicz.it (G.F.); eleonoravecchio@unicz.it (E.V.); nancynistico@unicz.it (N.N.); quinto@unicz.it (I.Q.); 2 Department of Pharmacy, Nutritional and Health Sciences, University of Calabria, Arcavacata di Rende, 87100 Cosenza, Italy; rossella.russo@unical.it; 3 Structural and Molecular Microbiology, Structural Biology Research Center, VIB, 1050 Brussels, Belgium; antonella.fioravanti@vub.be; 4 Structural Biology Brussels, Vrije Universiteit, 1050 Brussels, Belgium

**Keywords:** exosomes, cancer, liquid biopsy, tumor circulome, tumor monitoring, tumor derived exosomes hematological malignancies, minimal residual disease

## Abstract

Cells can communicate through special “messages in the bottle”, which are recorded in the bloodstream inside vesicles, namely exosomes. The exosomes are nanovesicles of 30–100 nm in diameter that carry functionally active biological material, such as proteins, messanger RNA (mRNAs), and micro RNA (miRNAs). Therefore, they are able to transfer specific signals from a parental cell of origin to the surrounding cells in the microenvironment and to distant organs through the circulatory and lymphatic stream. More and more interest is rising for the pathological role of exosomes produced by cancer cells and for their potential use in tumor monitoring and patient follow up. In particular, the exosomes could be an appropriate index of proliferation and cancer cell communication for monitoring the minimal residual disease, which cannot be easily detectable by common diagnostic and monitoring techniques. The lack of unequivocal markers for tumor-derived exosomes calls for new strategies for exosomes profile characterization aimed at the adoption of exosomes as an official tumor biomarker for tumor progression monitoring.

## 1. Introduction

In the common scenario, the cell is seen as a closed entity protected by its plasma membrane. Instead, the physiological and pathological functions in an organism depend on strong cellular connections. Cell-cell communication occurs among close and distant cells, involving different chemical signals, such as hormones, cytokines, signal molecules and proteins, and miRNAs, which are secreted by specific cell types. These signals exploit the circulatory stream and the extracellular spaces reaching their final destination by binding to specific receptors on the target cell surface or being internalized, activating a signaling cascade and producing several downstream effects.

**Figure 1:**
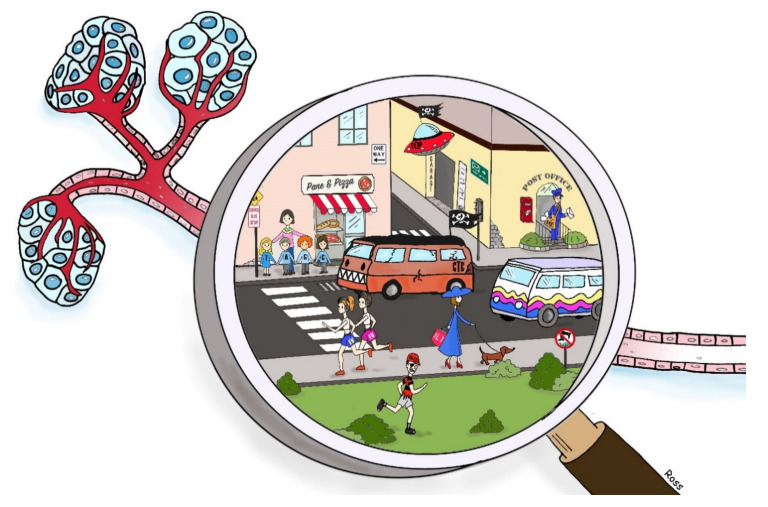
Cartoon representation of cancer circulome. Imagine observing a vessel coming from a tumor with a special magnifying glass. The vessel would appear as a lively neighborhood with the circulome components as main characters living their usual day. In front of a bakery and pizza restaurant, DNA nucleotides are pictured as kids accompanied by their teacher that are about to cross the road; they must pay attention to the red van (i.e., circulating tumor cell, CTC) that does not respect the highway code, unlike the colorful van (i.e., circulating cell, CC) that travels the road in peace. From the post office, the postman (i.e., mRNA) is ready to carry messages and letters, while on the other side of the road two young girls (i.e., extracellular vesicles, E.V.s) are doing a fast run on the sidewalk near an elegant lady in a blue dress (i.e., Interleukin-1) that is walking slowly with her little dog. On the lawn, in spite of the prohibitions, a bad looking guy (i.e., Tumor Derived Exosome, TDE) is running. Floating in the air, above all, an alien spacecraft (Tumor educated Platelet, TEP) mysteriously keeps track of the flow of time in this busy crossroad.

The ensemble of circulating components responsible for cell-cell communication is included in the concept of “circulome”. In pathological conditions, the circulome components reflect the course of a certain disease. The “tumor circulome” is constituted of circulating components derived from tumor tissues that includes circulating tumor cells, tumor derived nucleic acids, tumor-educated platelets, and a large subset of extracellular vesicles (Figure 1). In cancer biology, the tumor circulome is important for tumor monitoring, prognosis profiling, and diagnosis contribution [1].

## 2. Circulome and Liquid Biopsy

Tumors are biological entities that are constantly changing. The tumor dynamism is associated with the evolution of a delicate “tumor ecosystem”, which is composed by several interacting players that move from the primary cancer site up to long distances, where they trigger the formation of tumor niches, thus sustaining the progression of cancer metastasis and the relative tumor microenvironment (TME). The diagnostic techniques that are commonly used in oncology allow tumor diagnosis (e.g., tissue biopsy) and assessment of disease progression and therapy response; however, they are inevitably invasive and risky for the patient [2]. Thus, over the years, it has become necessary to study the tumor circulome by using less-invasive practices through an easy peripheral blood collection, according to the new approach named “liquid biopsy”. Indeed, the liquid biopsy analyzes the components of the tumor circulome in the peripheral blood, urine, liquor, and other body fluids, without the invasiveness of tissue biopsy. In particular, liquid biopsy can provide notable information on the tumor molecular features, unraveling details about the tumor progression in real-time and aggressiveness, as well as the therapy response [3].

Despite the standard techniques, such as Positron Emission Tomography - Computed Tomography (PET-TC) imaging and tissue biopsy, represent the gold standard in cancer diagnosis, the analysis of tumor circulome by liquid biopsy is a promising tool for early detection and follow up of cancer patients. However, there are some limitations in the choice of the circulome components as markers of tumor diagnosis, monitoring, and prognosis, as summarized in Table 1.

As far as the best candidate for diagnosis, it is not easy to demonstrate the reliable diagnostic utility of each circulome component. For example, Germano et al. assessed the difference in the use of Circulating Tumor Cells (CTCs) and circulating tumor DNA (ctDNA) in Liquid Biopsy of Colorectal cancer (CRC) patients [4]. CTCs were evaluable in one-third of CRC patients, while ctDNA was detected in all patients [4]. The first limitation of this method is the fact that the number of CTCs available, and thus detectable, in the patient is low. Such trademark represents a main difficulty in establishing the diagnosis. On the contrary, exosomes can be detected in huge quantities, almost always representing an indication of tumor activity. However, while the study on exosomes is able to contribute in small steps to the molecular evaluation of the tumor, CTCs analysis would provide a well-detailed scenario of tumor heterogeneity, being themselves directly belonging from primary tumor tissue. However, the low numbers of CTCs do not allow an error-free evaluation [5]. The association of the analysis of CTCs and Tumor Derived Exosomes (TDEs) could instead be promising, given the difficulties of the two approaches in diagnosis. Indeed, Etienne Buscail et al. evaluated the combined use of CTCs and exosomes for the diagnosis of pancreatic tumors. The huge advantage of liquid biopsy is in fact to use combined biomarkers to provide information in a rapid and non-invasive way [6].

With regard to the use of all circulome components in the liquid biopsy approach, it must be considered that the presence of circulome components does not represent an unequivocal sign of tumor presence, and tissue biopsy and radio-imaging techniques are still required for the final diagnosis [7].

## 3. Exosomes: The Smallest Extracellular Vesicles

After the outstanding discovery of micro-vesicles as exfoliation of the ectoenzyme membrane by Eberhard G. Trams and colleagues in 1981 [8], the term “exosome” was used for the first time a few years later by Pan and Johnson, when referring to small endosome-derived vesicles [9]. Exosomes were produced and secreted by sheep reticulocytes during their maturation process while eliminating transferrin receptors (Tfr) as trash proteins [9]. Later, it was demonstrated that these nanovesicles could have an important role as mediators of physiological pathways, especially pathological processes.

Classified as small E.V. of endosomal origin, the exosomes are 30–100 nm diameter vesicles, which are physiologically produced by all cell types and secreted through an exocytosis process in blood, urine, cerebrospinal fluid, other body fluids, and the medium of in vitro cell cultures. Even though the mechanisms of synthesis and secretion of exosomes have not been completely clarified, the crucial roles of the Endosomal Sorting Complex Required for Transport (ESCRT) and the intracellular Ca^2+^ increase are commonly recognized to be required for the exosomes release [10]. Exosomes play an important role in cell-cell communications. In fact, the exosomes reach very distant target cells, in which they release their cargo, represented by proteins, DNA, RNA, microRNAs, cytokines, and lipids, influencing both physiological and pathological processes. The exosomes isolated from different tumor cell lines selectively target the tissues of origin when inoculated in animals. It has been observed, in fact, that exosomes isolated from liver cancer-targeted the liver in vivo [11,12], while exosomes isolated from diffuse large B-cells Lymphoma (DLBCL) directly reached DLBCL cells [13]. Furthermore, the exosomes produced by esophageal cancer cells targeted parental cells, contributing to aggressiveness by promoting invasion and migration through a continuous transfer cycle [14].

The main question is how the exosomes recognize the target cells. To date, this issue has not yet been clarified, and several studies are focusing on the preferential internalization of exosomes in some cell types instead of others. Some reports identified specific molecules on the exosomal membrane and the membrane of target cells that guide the membrane-membrane interaction, resulting in the fusion of the membranes followed by the pouring of the exosomal cargo inside the target cell [15]. In particular, the expression of adhesion molecules and lipids on the exosomes surface seems to be fundamental for the binding to the target cell. Indeed, all exosomes express on their membrane surface the tetraspanin family members (CD63, CD81, CD9), Tsg101, Alix, MHC molecules, and HSP70 [16,17]. The secreted vesicles presumably encode the signal needed to target the recipient cell and to deliver the cargo through endocytosis or direct fusion with the cell membrane [18]. The exosomes that express ITGαvβ5 bind Kupffer cells and those that express ITGα6β4 and ITGα6β1 are able to bind fibroblasts and epithelial cells resident in the lungs, mediating, respectively, liver and lung tropism [11]. According to Rana et al., differences in tetraspanin complexes on the membrane are likely to influence the selection of target cells, both in vitro and in vivo [19]. Even more, the variety of tetraspanins on the exosomal membrane has the possibility of modulating associated proteins, including adhesion molecules [20]. In other cases, the cargo is released externally, and there is no interaction between the membranes or fusion mechanisms. It is, above all, the case of proteins and signal molecules, capable of binding the receptor expressed on the surface of the target cell in order to trigger that downstream signaling [21]. The exosomes’ production and content depend on the cell of origin, with a high difference between healthy and tumor cells. For these reasons, tumor-derived exosomes represent an optimal biomarker for monitoring tumor progression and minimal residual disease.

## 4. Exosomes Isolation

A large variety of protocols and a number of commercial kits are currently available for isolating exosomes and analyzing the exosome cargo [22]. Exosomes can be purified from serum, various biological fluids, or cellular medium by several centrifugations and ultra-centrifugations in a density gradient of sucrose. Alternative methodological approaches have been developed to overcome these labor-intensive and time-consuming methods. Filtration through specific cut-off engineered membranes, named “size-based filtration”, guarantees a more rapid protocol than ultracentrifugation, but part of the exosomes may be potentially deformed or damaged, negatively affecting the loss of molecules expressed on exosomes surface [23]. Exosomes can also be isolated by immunoaffinity chromatography, using antibodies against the exosome markers CD63, CD81, CD82, and CD9. These antibodies are immobilized on different supports, such as magnetic beads on plates of microfluidic devices or chromatography matrices. In addition to the common exosomes isolation techniques, several innovative microfluidic techniques have been recently developed, including the deterministic lateral displacement (DLD) sorting, trapping on nanowires, and acoustic nanofiltration [24,25]. Then, electron (SEM and TEM) or atomic force microscopy (AFM) are necessary to analyze the nature of the single isolated vesicle [26]. In addition to the common AFM or electron microscopy, other methods are in use to validate the exosomes populations quality, such as Nanoparticle Tracking Analysis, Dynamic Light scattering coupled with Zeta potential determinations, Western blotting, and other methods requiring antibodies labeling, such as fluorescence-activated cell sorting (FACS) and enzyme-linked immunosorbent assay (ELISA) (Table 2).

## 5. The Role of Exosomes in Solid Tumors

Several reports support the pivotal role of exosomes in tumorigenesis, mediating the immune response, antigen presentation, cell migration, cell differentiation, tumor survival, tumor invasion, and angiogenesis [27]. It was recently demonstrated that exosomes play a crucial role in metastatic events stimulating TME [28], transforming neighboring cells, and promoting secondary neoplasms [29]. Cancer cells migrate away from the primary tumor and settle in a new body district by establishing a tumor niche supported by chemical mediators of inflammation, which in turn help to recreate the tumor microenvironment [30].

In this context, the components of innate immunity play a particular role, such as polarized M2 macrophages, capable of releasing pro-angiogenetic factors contributing to the self-renewal of the tumor and its relapse [31]. Under hypoxic conditions, cancer cells are able to modulate the microenvironment in order to enhance angiogenesis, resulting in an increase in metastatic potential [32]. EGFR-derived exosomes can adjust the liver microenvironment, thus promoting the formation of gastric cancer metastasis [33]. The role of exosomes in the formation of the tumor vessels has recently been hypothesized [34]. For example, glioma exosomes contain pro-angiogenic factors that can contribute to tumor vascularization [35]. Their role in neovascularization is carried out, thanks to crosstalk with endothelial cells. TDEs are, in fact, able to modulate endothelial cells, contributing to their proliferation and migration [36]. A markedly decreased expression of inflammatory markers was found in Glioma patients’ exosomes, compatible with the effects of tumor-mediated immunosuppression [37].

Exosomes are capable of transferring metastatic potential to surrounding cells through the transfer of genetic information and/or pro-metastatic proteins [38]. Exosomes promote tumor growth and progression through a mechanism of inhibition of the immune system. In particular, several data show how exosomes are able to induce apoptosis of cytotoxic T-cells, inhibit the cytotoxicity of natural killer (N.K.) cells, or inhibit the differentiation of dendritic cells (D.C.) [39].

Interestingly, exosomes also induce drug-resistance in tumor cells due to their cargo in terms of multi-drug resistant protein release, tumor immune escape mechanisms, changes in apoptotic homeostasis, and tumor stroma interaction [40].

It has been shown that the treatment of a weak melanoma cell line (F1) with exosomes derived from the highly aggressive melanoma B16 cell line and with strong metastatic power, resulted in the expression of the metastatic marker met 72 in the F1 cell line, differently from untreated cells, showing that exosome treatment increased aggression of the weaker cell line [41]. In patients, melanoma derived exosomes were enriched in immunosuppressive proteins and inhibited CD69 expression, induced apoptosis, suppressed proliferation in CD8+ T cells, and down-regulated NKG2D expression in N.K. cells [42].

Proteomic analysis of metastatic melanoma derived exosomes identified proteins involved in cell motility, angiogenesis, and immune response. In particular, proteins involved in angiogenesis, such as EGFR, PTK2/FAK1, EPHB2, and SRC, have been found in metastatic melanoma cells derived exosomes, while proteins involved in the regulation of apoptosis and cell motility are represented in exosomes derived other melanoma weaker cell line, characterized by minor invasion capacity. Constantly, exosomes secreted by metastatic cell lines are able to increase the migratory capacity of less aggressive cells [43].

Treatment of some cancers requires therapies with immune checkpoint inhibitors, such as PD-L1, targeting antibodies [44]. Exosomes derived from cutaneous malignant melanoma (CMM) patients express PD-L1 on their surface, in the same way as exosomes derived from CMM cell lines [45]. Furthermore, the presence of PD-L1 is confirmed in 100% of the liquid biopsies performed while in only 67% of the tissue biopsies. PD-L1 on the surface of the exosomes could be used as a marker in monitoring the response to therapy in CMM [40]. Integrins expressed on exosomes surface are of considerable importance, able to promote the aggressive phenotype in many types of cancer, inducing both tumor invasion and migration. Tumor-derived exosomes express a particular pattern of surface molecules and Integrins repertoire able to bind several cell types and ECM molecules [46].

The Avβ3 integrin is part of the protein cargo of exosomes derived from prostate cancer (PoC) cell lines and has also been found in the blood exosomes of prostate cancer (PCa) patients. Its levels are higher in patients’ tumor-derived exosomes than in healthy donors [47]. Plasma Exosomes from prostate cancer patients are enriched in PSA, useful in distinguishing PCa patients from benign prostatic hyperplasia (BPH) patients [48].

CD151, CD171, and tetraspanin 8 were highly expressed in Non-Small Cells Lung Cancer (NSCLC) patients exosomes with respect to healthy donors, representing a powerful biomarker of tumor burden [49]. CD63 and caveolin 1 (CAV1) expressing exosomes were analyzed in stage IV oral squamous cell carcinoma (OSCC) patients [50]. In oral cancer, an increase in CAV1 expression represents a marker of tumor progression. CAV1 expressing exosomes increased in the case of tumor resection and decreased after a week post-resection [50].

Reflecting genetic and nongenetic cancer cell components, exosomes could contain miRNA with several regulation functions [51]. miRNAs were found useful in colon-rectal cancer (CRC) surgery success prediction. In fact, the expression of miR-200 and miR-14 in CRC resected patients’ blood exosomes correlated with overall survival (O.S.) [52]. miRNA-373 levels in triple-negative breast cancer (TNBC) patients’ serum exosomes were significantly increased in comparison with luminal tumors patients and healthy donors [53]. Hannafon and colleagues found a higher level of miR-1246 and miR-21 in breast cancer (BC) cell lines compared to mammalian control cell lines [54]. High levels of miR-1246 and miR-21 were found in patient derived xenografts (PDX) mice plasma exosomes and in plasma exosomes of BC patients compared to healthy donors [54]. Exosomal miR-1290 and miR-375 were found highly expressed in castration-resistant prostate cancer (CR-PCa), correlated with the worst patient prognosis [55]. In functional experiments, miR146a-5p, found in NSCLC patients, inhibits cell growth and migration and apoptosis induction of cancer cells [56]. Patients with advanced NSCLC with low miR-146a-5p levels in serum exosomes are characterized by higher recurrence rates. Furthermore, in the case of drug resistance during NSCLC therapy, the expression of miR-146a-5p is decreased as in the cases of advanced patients [57].

## 6. A Focus on Hematological Malignancies

Due to their ability to reach target cells located in far districts, the exosomes play an important role in the context of the hematopoietic system as they promote the crosstalk of different cell populations of bone marrow with the peripheral tissue. In order to support tumor survival and progression, hematological malignancies are characterized by the production and secretion of exosomes. Leukemia- or multiple myeloma-derived exosomes are involved in different processes and mechanisms, which promote drug-resistance, stimulation of tumor microenvironment, inhibition of the immune system, and re-modeling of the bone marrow [58,59].

In the last few decades, exosomes have generated great interest as a new powerful tool for the rapid and early prognosis of lymphoproliferative diseases. In 2014, Hong and colleagues revealed high plasma levels of exosomes (CD34high, CD33high, and CD117high) in acute myeloid leukemia (AML) patients compared to healthy controls [60]. Despite a significant reduction of tumor exosomes into plasma after chemotherapy, their concentration considerably increased in the course of minimal residual disease even when the leukemic blasts were not yet detectable. This evidence suggests that plasma levels of exosomes may contribute to the prognosis of leukemia [60]. Some years later, it was reported that the increased number of circulating exosomes correlated with their activity, highlighting the immunosuppressive activity of AML-derived exosomes, which interfered with anti-leukemia functions of immune cells, thus promoting tumor survival and progression [61].

Caivano et al. [62] also described the release of tumor exosomes in hematological cancer patients. They isolated E.V.s from the sera of different hematological cancer patients, such as chronic lymphocytic leukemia (CLL) and acute myeloid leukemia (AML), Hodgkin’s (H.L.) and non-Hodgkin’s lymphoma (NHL), and multiple myeloma (MM). They found higher levels of tumor exosomes in patients compared to healthy donors, strengthening the hypothesis on the potential role of exosomes for tumor monitoring [62]. Alongside the different number of exosomes between patients and healthy donors, another important feature of tumor exosomes is their cargo, including nucleic acids, proteins, and cytokines. In fact, the exosomes-dependent functions are carried out once the exosomes content has been poured into the target cell. In the course of the disease, exosomes could support the tumor evolution (e.g., from indolent to aggressive stage), as well as stimulate or inhibit accessory cells in the TME. By analyzing the proteomic profiles of plasma exosomes of CLL patients at different stages of the disease, Prieto et al. reported higher levels of S100-A9 protein in tumor exosomes from patients with progressive disease [63]. Moreover, exosomes with S100-A9 cargo were able to enhance the NF-κB activity in CLL cells from indolent patients [63].

Well known is the role of monocytes, macrophages, and innate immunity components in the progression of neoplasms, mediated by the action of cytokines and interleukins [64]. The CLL-exosomes-mediated reprogramming of TME through the transition of stromal cells to cancer-associated fibroblasts had a powerful repercussion on the progression of the disease [65]. CLL-derived exosomes were rich in small miRNAs, such as Y RNA hY14, a particular miRNAs family that is normally rare in the blood of CLL patients and healthy donors [66]. The plasma exosomes of CLL patients mainly expressed the surface markers CD37, CD63, and CD9, but not CD3, CD56, and CD41, suggesting the genesis of these exosomes from B-lymphocytes and not from T-lymphocytes or other blood cells [67], confirming the promising role of exosomes in monitoring hematological tumors. Although CD41 is a marker of activated platelets, it has been reported that a minimal fraction of total plasma exosomes is derived from platelets [68]. Yeh et al. evaluated the presence of tumor exosomes in CLL patients and healthy subjects and assessed the expression of the exosomal miRNA profile, identifying up-regulated miR-150, miR-155, and miR-29, as well as down-regulated miR-223. These miRNAs are always associated with CLL pathogenesis [67]. Moreover, exosomal miRNAs could be responsible for the evolution of CLL in Richter’s syndrome, according to Jurj and colleagues, who provided the evidence that miR-19 was the most abundant component of patient exosomes [69]. miR-19 up-regulated Ki67 and down-regulated p53, leading to apoptosis escape and CLL evolution in Richter’s syndrome [69].

Shifting the focus from CLL to MM disease, MM-derived exosomes showed a strong component in miRNAs. It is well known that circulating miRNAs, whether present or absent in exosomes, have a pivotal role in tumorigenesis. In particular, let-7b and miR-18a were found in exosomes derived from MM patients and acted as independent predictors of survival and progression-free survival [70]. In 2019, Zhang et al. identified a potential relationship between the presence of exosomal miRNAs and their clinical significance in MM patients [71]. In particular, they showed a lower expression of let7d miRNA in MM patients with respect to healthy donors. miR-let7-d is a negative regulator of Interleukin (IL)-6. Of note, the low expression of miR-let7-d positively affected IL-6 levels and worsened the MM patients’ prognosis and overall survival [71]. Several other papers discuss the role of exosomes in increasing the aggressiveness of certain cancer types based on proliferation assays, cell viability, and response to drug tests. The fact that exosomes influence tumor aggressiveness is a clear signal of their action in promoting the tumor switch toward drug-resistance and apoptosis escape [72]. Indeed, it was shown in drug-resistant MM cells that exosomes derived from bone marrow stromal cells (BMSC) are responsible for the increased cell proliferation and resistance to bortezomib treatment, suggesting a key role of BMSC-derived exosomes in MM aggressiveness [73].

The role of exosomes was also analyzed in mantle cell lymphoma (MCL), which requires new methods for monitoring due to the rarity and poor prognosis of this disease. A study by Hazan-Halevy et al. demonstrated the major presence of MCL exosomes in patients with high white bloos cells (WBC) count [74]. Moreover, they showed that MCL exosomes specifically reached B-lymphocytes, where they were internalized and released the cargo of lipid raft/cholesterol by the endocytosis-driven pathway [74]. Further, it is well demonstrated that CD19+ exosomes derive from B-cells, and this also applies to B-cell lymphomas, acute lymphoblastic leukemia (ALL), and CLL. Additional data indicated that DLBCL-derived exosomes expressed the canonical endosomal markers (Alix, CD81, TSG101, and CD63) and the B-cell marker CD20 [75]. However, conventional diagnosis of B-cell malignancies is based on the up- or down-expression of several biomarkers, such as CD5 [76], CD34 [77], CD123 [78], CD20 [79], and CD138 [80].

## 7. Discussion

The invasive nature of traditional biopsy is an unfriendly procedure and can lead to a misleading cancer profiling and poor monitoring of tumor progression due to the genetic and phenotypic intra-tumor heterogeneity [81,82,83]. Representing the molecular footprint of the parental cell, exosomes and especially TDEs has been recently taken into consideration in order to design a reliable liquid biopsy for non-invasive monitoring of tumor evolution and recurrence, and useful to evaluate the therapy response [84,85]. The lipid bilayer membrane confers stability, preventing the exosomal cargo from degradation circulating throughout the body. Because of this stability, exosomes are easily harvested from a variety of accessible body fluids. This makes them attractive targets for developing new methods for detecting cancer [86]. While the last decades have been characterized by the development of protocols for efficient exosomes purification [87] (including commercially available reagents [88]), the major challenge yet to solve is the so-called “Rubik’s cube” that is represented by figuring out the exosomes’ complexity. The extracellular vesicles are a very heterogeneous whole with considerable diversity based on the origin, morphology, size, and, finally, the nature of the content [89]. In the beginning, it was thought that exosomes were a fairly homogeneous class of extracellular vesicles, but more recent experimental pieces of evidence have highlighted the enormous diversity in the exosome category so as to define distinct subtypes released for example from the apical or basolateral surface of tumor cells [90]. The various subtypes of exosomes differ according to biogenesis [91], lipid composition [92], surface markers [93], and obviously for the cargo [94].

A clear evidence is that the presence of a larger quantity of exosomes detected in cancer patients compared to healthy individuals represents a clear sign of tumor presence and progression. However, this is not enough to clinically validate the exosomes as excellent biomarkers for tumor monitoring since we need to identify specific markers of exosomal subtypes and to characterize their peculiar function and/or group of functions relatives to the tumor progression.

Efforts in this direction have been made by researchers worldwide applying different approaches and opening up new avenues in devising liquid biopsy options for effective early detection of TDEs shed into the body fluids by tumor cells. A basic approach to unravel the correlation between cancer progression and exosomes is represented by the evaluation of differential expression of common exosomal surface proteins in pathological conditions. In this context, Yuh-Ying Yeh and co-workers have identified the tetraspanin, CD9, CD63, and CD37 as abundantly expressed markers in tumor exosomes of CLL patients by flow analysis and immunoblotting, in contrast to the modest expression of CD41, CD56, and CD3 [67].

By reflecting the proteomic and lipidomic profile of their parental cells, exosomes can be validated by mass spectrometry for the presence of tumor markers overexpressed by their tumor of origin. In 2017, Alicia Llorente and colleagues performed a high-throughput mass spectrometry quantitative analysis of urinary exosomes of prostate cancer patients and healthy controls and compared them with those produced by prostate cancer cell lines [94]. They proved the potential use of lipids as diagnostic tools for prostate cancer since the alterations of exosomal lipid components were comparable to the alterations in lipid metabolism in the parental cancer cells [95]. Several studies questioned the role of exosomes in modulating the tumor microenvironment of hematological tumors. In particular, Haderk et al. discovered CLL exosomes containing an miRNA family in a greater quantity compared to other miRNAs in the parental cells, suggesting that CLL cells continuously pour into the circulation exosomes that are enriched of peculiar functions required for TME evolution. Consistently, the treatment of MEC-1 cells with the hY14 miRNA increased the cytokines and interleukins released from monocytes and immunosuppressive molecules [66]. This evidence raises several questions on the role of tumor-derived exosomes as accessory components required for cancer evolution by acting at the level of the tumor microenvironment and surrounding cells. More effort should be put into clarifying the mechanisms of preferential packaging of molecules as cargo of the extracellular vesicles, with the purpose of proving their influence on tumor microenvironment and their effective role as tumor progression markers.

An additional approach for personalized medicine is the development of antibodies and peptides, recognizing biomarkers that are unequivocally expressed by tumor-derived exosomes. In the context of B-cells malignancies, a key role is played by the immunoglobulin B-cell receptor (IgBCR). The IgBCR is expressed on the surface of the B-cell membrane surface during the B-cell development, and it is responsible for antigen-binding and specific immune response. Several reports demonstrated the role of IgBCR in the pathogenesis and progression of B-cells malignancies, such as CLL [96] and MM [97], through altered signaling [98,99]. As the IgBCR expresses a specific antigen-binding site, it can be considered a tumor B-cell biomarker in the case of B-lymphoproliferative disorders. In this regard, Iaccino and colleagues demonstrated that the exosomes isolated from 5T33MM cell line medium expressed the canonical exosome markers and the same antigen-binding site of the IgBCR of parental MM cells [100]. This result indicated that the IgBCR expressed by 5T33MM cells was a specific marker of 5T33MM-derived exosomes, revealing it to be a sensitive and not invasive tool for monitoring the tumor progression in 5T33MM-xenografted mice [99]. Peptides recognizing specific biomarkers of tumor exosome membrane could be useful for the isolation of the extracellular vesicles, thus solving most of the problems deriving from the equivocality of the normally known exosome markers. Regarding the use of peptides to select populations of exosomes, Marina Cretich and co-workers have recently developed a promising and low-cost method that uses membrane-sensing peptide ligands to bind the extracellular vesicles, avoiding the use of antibodies and allowing a good yield [101].

**Table 2:** Overview of literature highlighting the exosomes isolation and characterization approaches in cancer research.

Malignancies	Exosomes Isolation Method	Exosomes Characterization	Comments	Ref.
**Acute Myeloid Leukemia**	Sucrose gradient centrifugation, Size exclusion chromatography	Transmission electron microscopy-Western Blotting-Nanoparticle Tracking Analysis Fluorescence-activated cell sorting	Exosomal proteins TGFβ_1_ and TGFβ Increase in MRD course of CD34, CD33, and CD117 exosomes when leukemic blasts are not detectable	[60]
	Size exclusion chromatography on Sepharose column	Western Blotting-Tunable resistive pulse sensing-Transmission electron microscopy	Effect of TGFβ exosomes on NK92 cells	[61]
	Centrifugation	Fluorescence-activated cell sorting	Surface markers and different exosomes concentration in sample patient	[62]
**Chronic Lymphocytic Leukemia**	Centrifugation	Nanoparticle Tracking Analysis	JUP, S100-A9, and exosome’s proteome profiling in CLL evolution	[63]
	Centrifugation	Nanoparticle Tracking Analysis ELISA-UPLC-Mass spectrometry	Exosomal miRNA distribution and its effect in generating a tumor supportive microenvironment	[66]
	Centrifugation	Fluorescence-activated cell sorting	Surface markers and different exosomes concentration in sample patient.	[62]
	Centrifugation	Nanoparticle Tracking Analysis Fluorescence-activated cell sorting	Surface antigens and correlation with BCR signaling and miRNA profiling.	[67]
	Centrifugation, Filtration, Sucrose density centrifugation	Finite track length adjustment Transmission electron microscopy	Prediction of the CLL evolution in RS.	[69]
**Multiple Myeloma**	Exosome isolation reagents	Abs labeling-Transmission electron microscopy-Nanoparticle Tracking Analysis	Exosomal miRNA let7b and let18-a in monitoring the disease.	[70]
	Exosome isolation reagents	Western Blotting	miRNA profile and clinical features of MM	[71]
	Exosome isolation reagents	Nanoparticle Tracking Analysis Transmission electron microscopy	miRNA profile and clinical features of MM	[73]
	Centrifugation	Fluorescence-activated cell sorting	Surface markers and different exosomes concentration in sample patient	[62]
	Exosome isolation reagents	Env. Scanning Electron Microscope Dynamic light scattering Zeta potential determinations Western Blotting	IgBCR expressed on exosome surface. Exosomes in monitoring B cell disease.	[99]
**Non-Hodgkin Lymphomas**	Centrifugation	Fluorescence-activated cell sorting	Surface markers and different exosomes concentration in sample patient	[62]
	Centrifugation	Nanoparticle Tracking Analysis Fluorescence-activated cell sorting	Surface exosome markers characteristic of B-cells involved in DLBCL	[75]
	Exosome isolation reagent	Fluorescence-activated cell sorting Transmission electron microscopy Nanoparticle Tracking Analysis	Exosome internalization. MCL derived Exosome structural and biochemical characterization	[74]
**Hodgkin’s Lymphomas**	Centrifugation	Fluorescence-activated cell sorting	Diversity in exosomes surface markers and concentration in patients	[62]
**Breast Cancer**	Exosomes isolation reagents	Western Blotting	miRNAs profiling in TNBC	[53]
	Centrifugation Exosomes isolation reagents	Transmission electron microscopy Nanoparticle tracking analysis Western blotting	miRNAs profiling in BC	[54]
**Colorectal Cancer**	Centrifugation	Cryo Transmission electron microscopy-Western Blotting	miR-200c and miR-141 in MV plasma can identify CC patients with poor prognosis	[52]
**Oral Squamous Cell Carcinoma**	Centrifugation	Western Blotting	Monitoring stage 4 oral squamous cell carcinoma through exosomes detection	[50]
**Prostate Cancer**	Centrifugation, Sucrose density, Iodixanol gradient, Exosome reagents	Nanoparticle tracking analysis Transmission electron microscopy	Prostate cancer sheds the αvβ3 integrin in vivo through exosomes	[47]
	Centrifugation	Nanoparticle tracking analysis ELISA-Fluorescence activated cell sorting Western Blotting	PSA in Exosomes distinguish PCa patients from BPH	[48]
	Exosome isolation reagents	Nanoparticle tracking analysis	Exosomal miR-1290 and miR-375 as prognostic markers in CR-PCa	[55]
**Lung Cancer**	EVs MicroArray	EVs Micro Array Nanoparticle Tracking Analysis	CD151, CD171 and tetraspanin 8 were highly expressed in NSCLC	[49]
	Exosome isolation reagents	Transmission Electron Microscopy, Western blotting - Nanoparticle Tracking Analysis	Predictive value of exosomal miRNA in NSCLC	[57]
**Glioma**	Centrifugation Ultrafiltration	Transmission electron microscopy Nanoparticle tracking analysis	Glioma exosomes promote angiogenesis	[35]
	Centrifugation	Nanoparticle tracking analysis ELISA-Western Blotting	Exosomes-mediated immunosuppression	[37]
**Cutaneous Malignant Melanoma**	Centrifugation Sucrose density gradient	Fluorescence activated cell sorting Western Blotting	Exosomes transferring metastatic potential between melanoma cell lines	[41]
	Centrifugation, ultrafiltration, size exclusion chromatography	Nanoparticle tracking analysis	Immunosuppression CD8+ cells suppression Downregulation of NKG2D NK cells	[42]
	Centrifugation, sucrose density gradient	Silver staining-Western blotting Nanoparticle Tracking Analysis, Transmission Electron Microscopy	EGFR, PTK2/FAK2, EPHB2, SRC Expression in exosomes	[43]
	Centrifugation	Nanoparticle tracking analysis	Presence of PD-L1 on exosomes surface	[45]

AML: Acute Myeloid Leukemia; CLL: Chronic Lymphocytic Leukemia; MM: Multiple Myeloma; NHL: Non-Hodgkin’s Lymphoma; HL: Hodgkin’s Lymphoma. DLBCL: Diffuse Large B-cells Lymphoma; MCL: Mantle cell Lymphoma; RS: Richter’s Syndrome; BCR: B-cell receptor; TLR: Toll-like receptor; MRD: minimal residual disease. TNBC: Triple Negative Breast Cancer. BC: Breast Cancer. CR-PCa: Castration Resistant Prostate Cancer. NSCLC: Non-Small Cell Lung Cancer. BPH: Benign prostatic Hyperplasia.

## 8. Conclusions

The exosomes biology and function strongly indicate their potential applications for non-invasive tumor diagnosis and monitoring. Based on the experimental results on TDE-associated with cancer research, we need further insight into the molecular nature of exosomes and the distribution of some categories of exosomes containing supportive tumor molecules. The final goal is to detect exosomes by using biomarkers matching the most representative signatures of the parental tumor cells. Recent progress in exosomes isolation by nanotechnology techniques represent the cornerstones in the device of cost-effective exosome-based diagnostics that will hopefully allow a full exploration of the potential application of TDEs in the clinical practice for cancer diagnosis and beyond.

## Figures and Tables

**Table 1:** Source and clinical use of circulome components [4,5,6,7].

Circulating Biomarkers	Source	Early Stage Detection	Final Diagnosis	Progression Monitoring	Prognosis Profiling
**CTCs**	Blood, ascites, saliva, urine	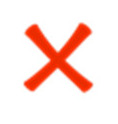	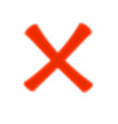	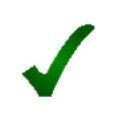	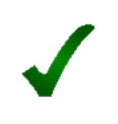
**ctDNA**	Blood, urine, pleural effusion, saliva, CSF	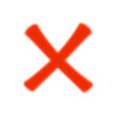	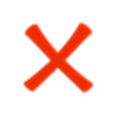	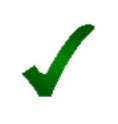	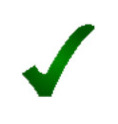
**ctRNA**	Blood, urine, pleural effusion, saliva, CSF	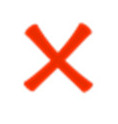	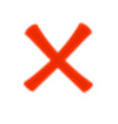	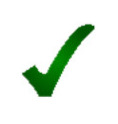	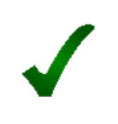
**TDEx**	Blood, Urine, Milk, BLF, Saliva, BAL	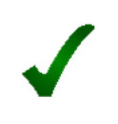	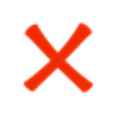	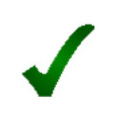	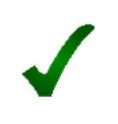
**TEPs**	Blood	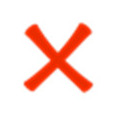	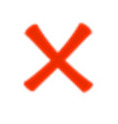	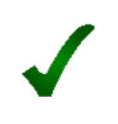	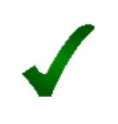

CTCs: Circulating Tumor Cells; ctDNA: circulating tumor DNA; ctRNA: circulating tumor RNA; TDEx: Tumor derived Exosomes; TEP: Tumor educated Platelets; BLF: Amniotic Fluid Bile; CSF: cerebrospinal fluid; BAL: bronchoalveolar lavage.
